# Inhibition of Human Cytomegalovirus Particle Maturation by Activation of Liver X Receptor

**DOI:** 10.3389/fmicb.2022.846386

**Published:** 2022-03-07

**Authors:** Bingnan Liu, Yanping Ma, Yujing Huang, Zhongyang Liu, Qiang Ruan, Ying Qi

**Affiliations:** ^1^Virology Laboratory, Shengjing Hospital of China Medical University, Shenyang, China; ^2^Department of Pediatrics, Shengjing Hospital of China Medical University, Shenyang, China; ^3^Department of Obstetrics and Gynecology, Shengjing Hospital of China Medical University, Shenyang, China

**Keywords:** human cytomegalovirus, liver X receptor, virus assembly compartment, virus particles, cholesterol

## Abstract

Human cytomegalovirus (HCMV), a herpesvirus family member, is a large, complex enveloped virus. The activation of liver X receptor (LXR) can significantly inhibit the replication of HCMV and weaken the virulence of progeny virus (unpublished data). Our results showed activated LXR affected some important viral protein expression and reduced cholesterol content in HCMV infected cells and virus particles. To further clarify the influence of activated LXR on HCMV replication, HCMV assembly and maturation processes were studied by transmission electron microscopy (TEM) in HCMV infected foreskin fibroblasts treated with LXR agonist GW3965. Results showed that activated LXR could reduce the envelope integrity of maturating virions. The functional stage of activated LXR on viral envelope integrity was mainly at virus assembly compartment (VAC) mediated envelopment but not structurally complete virus nucleocapsid formation and the egress of nucleocapsid from the nucleus to the cytoplasm mediated by nuclear egress complex. Reduced cholesterol synthesis and viral protein expression might interfere with the VAC-mediated envelopment. The nucleocapsid and tegument proteins enter the VAC area for the secondary envelope, which was interfered with and resulted in the defective particle, thereby affecting the amount and infectivity of the mature virus. The results indicate that inhibition of HCMV maturation is one mechanism of activated LXR inhibiting virus replication in infected cells.

## Introduction

Human cytomegalovirus (HCMV) is a large, complex enveloped virus that belongs to the herpesvirus family (Mettenleiter et al., 2009). HCMV infection causes severe diseases in immunocompromised individuals such as AIDS patients and organ transplant recipients and is the most common and harmful pathogen causing congenital infection and birth defects (Landázuri et al., 2021).

Our study (unpublished data) has shown that HCMV growth was remarkably inhibited by the activation of liver X receptor (LXR). Virus particles undergo a series of complex processes during packaging and maturation. Therefore, it was worth investigating how activated LXR affected the virus titers and infectivity of progeny virus.

In recent decades, it has been agreed that LXR-α acts as a cholesterol sensor, stimulating the transport of cholesterol in cells in the presence of excess cholesterol (Korach-André and Gustafsson, 2015). However, cholesterol is one of the important components promoting the stability of cell membrane structure, and the integrity of cytoplasmic and cell membrane seriously affects the viral envelope (Bilotta et al., 2020). Therefore, quantities of cholesterol in the progeny virus particles produced from cells treated with LXR agonist GW3965 were detected.

Human cytomegalovirus replication cycle involves nuclear and cytoplasmic stages. Viral capsid assembly and initial tegumentation all occur within the host cell nucleus, relying heavily on evolutionarily conserved viral proteins (Waddington et al., 2015). HCMV causes a major reorganization of cellular membrane organelles to generate the virus assembly compartment (VAC) in the cytoplasm, which is unique in beta-herpes virus-infected cells. Virus particles congregate in the VAC during late phases of infection, consistent with its significant role in controlling final tegumentation, envelopment, and egress from cells. The processes of HCMV assembly and maturation were studied by transmission electron microscopy (TEM) in HCMV infected human foreskin fibroblasts (HFF) treated with LXR agonist GW3965 to understand the influence of activated LXR on the progeny virus particles.

## Materials and Methods

### Cells and Virus

Human foreskin fibroblast (HFF) cells were grown in minimal essential medium (MEM) (Biological Industries) containing 10% fetal bovine serum (FBS) and 1% penicillin-streptomycin solution and incubated in Steri-Cycle CO_2_ Incubator (Thermo Electron Corporation) containing 5% CO_2_.

Human cytomegalovirus Towne strain was prepared by ultra-centrifugation from a bacterial artificial clone (BAC) that is a present of Yongjun Yu in the United States and was stored at −80°C. The Towne strain contained a green fluorescent protein gene. The infection titer of the stock was titrated as 4.6*10^7^ PFU/ml by 50% tissue culture infective dose (TCID50) as described below.

### Preparation of Study Samples

The HFF cells (1*10^7^) were synchronized with FBS free medium for 24 h before the treatments. Mock infected and HCMV infected HFFs treated with or without LXR agonist GW3965 were prepared. For the LXR-activated group, cells were treated with 2 μM GW3965 for 1 h before HCMV infection. Then, HFF cells in infection groups were inoculated with HCMV (Towne) at an MOI of 3. The cells were collected at 72 h post infection (hpi).

Human foreskin fibroblasts cells were inoculated with HCMV and treated with LXR agonist GW3965 as before. The progeny virions from cells in 72 hpi treated with or without GW3965 were purified by ultracentrifuge (optima XPN-80 ultracentrifuge, Beckman Coulter) with the centrifugal force 55,000 *g* for 1 h at 20°C.

### Western Blot

For Western blot, whole-cell extracts and virions were resuspended in phosphate buffer saline (PBS), then the pellets were harvested and lysed with cell lysis buffer containing protease inhibitor cocktail. Equal amounts of total protein in the cell lysates were mixed with sodium dodecyl sulfate (SDS) buffer and were boiled at 95°C for 10 min. The proteins were separated by electrophoresis in 10% polyacrylamide gels and blotted onto PVDF membranes (catalog number IPVH00010; Merck). Membranes were sequentially probed with primary antibodies and appropriate peroxidase-conjugated secondary antibodies, excited using High-sig ECL Western Blotting substrate (catalog number 180-501; Tanon), detected using a ChemiDoc™ Touch Imaging system (732BR2203; BIO-RAD), and quantified by densitometry using ImageJ software (National Institutes of Health). The following antibodies of LXR alpha (ab176323; Abcam), anti-ex2/3, anti-pp28 (sc-56975; Santa Cruz), anti-pp52 (sc-69744; Santa Cruz), anti-pp65 (sc-52401; Santa Cruz), anti-gB (Mab13513, Abnova), and anti-GAPDH (Ab8245; Abcam) were used as primary antibodies in this study.

### Detection of Human Cytomegalovirus DNA in the Progeny Virions by Quantitative Polymerase Chain Reaction

The purified virus stock from cells treated with or without GW3965 was first 1:100 diluted as an initial sample. Viral DNA copies were detected by quantitative polymerase chain reaction (qPCR) using HCMV UL123 primers. qPCR was performed on a real-time thermocycler (Applied Biosystems: QuantStudio™ 5) using QuantiNova SYBR Green PCR Kit (Qiagen, 208054) in a 20 μl reaction. Reaction conditions were denaturation at 95°C for 2 min, followed by 40 two-step cycles of 95°C for 10 s and 60°C for 30 s. The HCMV UL123 primers are 5′ -GGGGTTCTCGTTGCAATCCT- 3′ and 5′- GAGTTGGCCGAAGAATC CCT- 3′. UL123 sequence: 5′ -GGGGTTCTCGTTGCAATCCTCGGTCACT CGTTCAAAAGTTTTGAGGGATTCTTCGGCCAACTC- 3′ was synthesized as UL123 quantitative standard. 10-fold serial dilutions of the UL123 quantitative standard were used to generate standard curves.

### Detection of Cholesterol in the Whole-Cell Extracts and the Progeny Virions

The Cholesterol/Cholesteryl Ester Quantitation Kit (Bio-Vision, Catalog: K603-100) was used to detect quantities of free cholesterol, cholesteryl esters by colorimetric methods. The Cholesterol Standard was diluted with Cholesterol Assay Buffer and added to wells generating 0, 1, 2, 3, 4, and 5 μg/well of the Cholesterol Standards, plotted the Cholesterol Standard Curve.

For the cell samples (72 hpi), 10^6^ cells could be extracted with 200 μl of chloroform: Isopropanol: NP-40 in a micro-homogenizer. Spin the extract in the centrifuge. Transfer all the liquid to a new tube, air dry to remove chloroform, and trace organic solvent. Dissolve dried lipids with 200 μl of Cholesterol Assay Buffer. The progeny virions (72 hpi) were diluted 10-fold in the Cholesterol Assay Buffer. After incubation at 37°C for 60 min in dark conditions, the absorbance of the samples was measured at 570 nm for the colorimetric assay, and the total cholesterol concentrations were calculated by comparing to those of the Cholesterol Standards.

### Titration of the Virions in 72 hpi

The HFF cells were seeded into 96-well dishes at a 2*10^5^ cells/ml concentration. The purified virus stock from cells and HCMV infected cells in 72 hpi treated with or without GW3965 was diluted to a series of concentrations. The cells were inoculated with the diluted virus and cultured in growth medium for approximately 14 days. The number of GFP-positive wells was accounted for under a fluorescent microscope. The corresponding titer of the virus stock was calculated using the TCID50 chart.

### Detection of Virions in Infected Human Foreskin Fibroblasts by Transmission Electron Microscopy

The HCMV capsid assembly and maturation processes were studied by TEM (HITACHI-H7650, accelerating voltage 80kV). HFF cells in 72 hpi were fixed with 2.5% glutaraldehyde for at least 2 h at 4°C and then were treated with 1% osmium tetroxide, dehydrated through a graded series of ethanol with concentrations (from 30 to 70%), and embedded with EPON 812 epoxy resin embedding agent for 24 h warming up from 35 to 60°C. Ultrathin sections (approximately 70 to 90 nm) of embedded specimens were prepared, deposited onto Formvar-coated copper grids, and double strained with Uranyl acetate and lead citrate, enhancing contrast. By TEM, a single HFF cell infected with HCMV was sought out at 80 kV.

### Statistical Analyses

The SPSS software was used for statistical analysis, and all the graphs were generated with Prism GraphPad software. All experiments were repeated at least three times, presenting the representative results. Data are presented as mean **±** standard deviation (SD) and were analyzed by a two-tailed unpaired *t*-test. The difference was considered significant if *P* <0.05.

## Results

### Activated Liver X Receptor on the Expression of Important Proteins in Human Cytomegalovirus Infected Human Foreskin Fibroblasts Cells and Progeny Virus Particles

**FIGURE 1:**
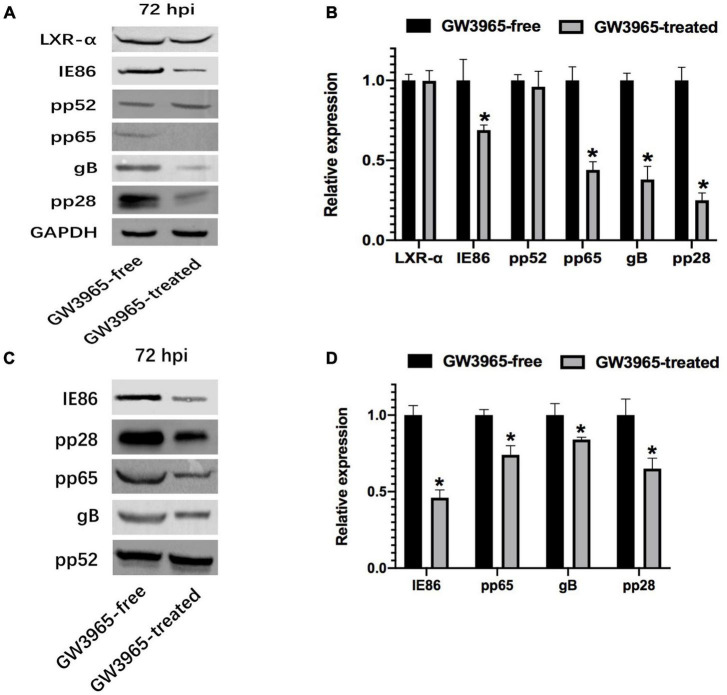
Effect of activated LXR on the expression of important protein. **(A)** Expression of important viral proteins was detected by Western blot in HCMV-infected HFFs. The cells were infected with HCMV Towne strain and treated with or without GW3965 for 72 h. GAPDH was used as a loading control of total proteins. LXR, IE, gB, pp65, pp28, and pp52 were detected. **(B)** The bands from three repeated experiments were scanned, the density of bands was quantified and the gray values of the two groups were compared. The ratios of gray values from each sample are used to conduct statistical analysis, *P* < 0.05 (*n* = 3). **(C)** Expression of important viral proteins was detected by Western blot in purified virions harvested from HCMV-infected HFFs treated as in panel **(A)**. **(D)** The pp52 was used as a loading control of virions. The ratios of gray values are treated as in panel **(C)**. *P < 0.05 (*n* = 3).

To clarify whether the activated LXR affects the expression of important proteins, LXR-α, IE, gB, pp65, pp28, and pp52 in infected cells and purified progeny virions were detected by Western blot. The protein level of LXR-α, which is inherent in host cells, was not obviously increased in the HFFs by the treatment of LXR agonist. Through comparative analysis of the experimental results, we found that the expression level of pp52, which is the accessory subunit of DNA polymerase, was not almost changed in cells and purified progeny virions from the cells whatever treatments with or without GW3965. The pp52, as an intrinsic component of HCMV, was used as loading control of virions in this study. Compared with GW3965-free cells, the relative gray values of various protein expression levels in GW3965-treated cells were 0.69 ± 0.03 (IE86), 0.38 ± 0.08 (gB), 0.44 ± 0.05 (pp65), and 0.25 ± 0.05 (pp28), respectively (Figures 1A,B). The relative gray values of various protein expression levels in the purified virions from the cells were 0.46 ± 0.05 (IE86), 0.84 ± 0.02 (gB), 0.74 ± 0.06 (pp65), and 0.65 ± 0.07 (pp28) (Figures 1C,D).

With GW3965 treatment, the expression of immediate-early protein (IE86) and envelope glycoprotein (gB) decreased in HFFs and virus particles. As important tegument proteins, the expression of pp65 and pp28 also declined in HFFs and virus particles after LXR activation.

### Effect of Activated Liver X Receptor on the Quantity of Human Cytomegalovirus Genome DNA in the Virions

The purified virus stock from cells was first 1:100 diluted as an initial sample. The viral DNA copy number of the purified mature virus particles from GW3965-treated cells was about 7.05*10^8^ copies/mL, and 1.76*10^9^ copies/mL in those from the untreated cells. Copy numbers of viral DNA in virions of 1:100 dilution treated with GW3965 decreased by about 2.5-fold.

### Effect of Activated Liver X Receptor on the Quantity of Cholesterol in Human Cytomegalovirus Infected Human Foreskin Fibroblasts and Progeny Virus Particles

The lipid quantity in the envelope of viral particles affects viral infectivity. To determine whether the inhibition on the virus titer of activated LXR comes from the decrease of lipid quantity in mature progeny virus particles, the cholesterol quantities both in the cells and the purified progeny virus particles of HCMV infected HFFs treated with or without GW3965 for 72 h were detected. Meanwhile, the virus titer of purified virus particles was detected, too.

**FIGURE 2:**
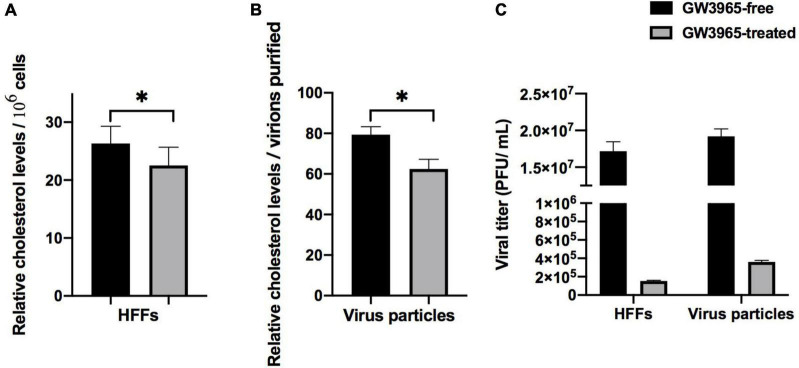
Effect of activated LXR on cholesterol quantity. **(A)** Activated LXR decreases cholesterol quantity in HCMV infected human foreskin fibroblast. **(B)** Relative cholesterol levels in virions. **(C)** The viral titers of cells and virus particles with and without LXR agonist GW3965 treatment have been provided *P < 0.05.

Based on the counting numbers of the detected cells, the cholesterol in the GW3965-free and the GW3965-treated cells was 26.7 ± 1.3 μg/10^6^ cells and 22.5 ± 1.8 μg/10^6^ cells, respectively. The cholesterol quantity of the cells decreased by 1.19 times (Figure 2A). The titer of GW3965-treated cells was 1.53*10^5^ PFU/mL, and that from the GW3965-free cells was 1.68*10^7^ PFU/mL (Figure 2C). The infectivity of the virus particles in cells decreased about 110 times after LXR activation.

The cholesterol quantity in mature virus particles purified from GW3965-treated cells similarly decreased compared to those of GW3965-treated cells. The cholesterol quantities of the virus particles extracted from the GW3965-free group and GW3965-treated group were 79.4 ± 4.3 μg/10^6^ cells and 62.1 ± 2.3 μg/10^6^ cells, based on the number of cells from which the virions purified, respectively. The cholesterol quantity of the progeny virus particles from cells treated with GW3965 decreased by 1.28 times (Figure 2B) to that from untreated cells. Correspondingly, the titer of the progeny virions purified from the GW3965-treated cells was 3.60*10^5^ PFU/mL, and that from the GW3965-free cells was 1.92*10^7^ PFU/mL (Figure 2C). The infectivity of the purified virus particles decreased about 53 times after LXR activation.

These results indicated that a decrease of cholesterol quantity in the GW3965-treated cells and virions resulted in defective envelopes and weak infectivity of the progeny virions.

### Effects of Activated Liver X Receptor on Viral DNA Encapsidation

**FIGURE 3:**
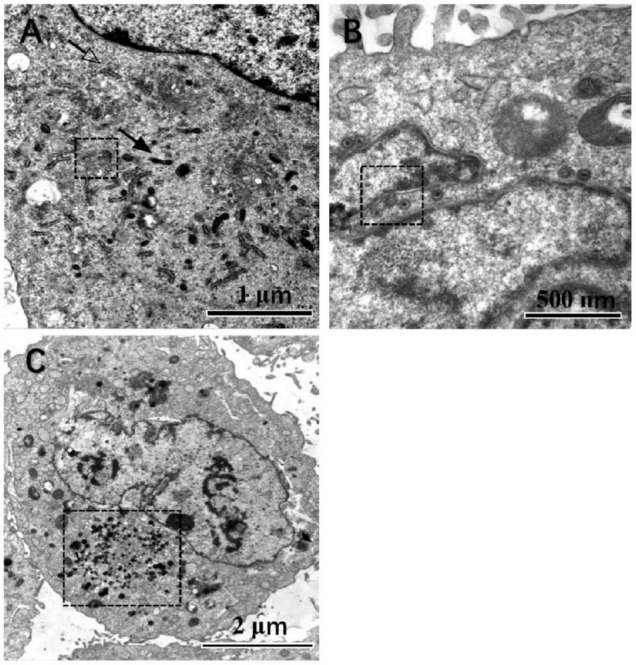
Cells observed under the TEM, magnification is marked. In mock-infected cells **(A)**, those pointed by the solid arrow and hollow arrow point are mitochondrion and endoplasmic reticulum, respectively. The organelle in the dotted line box is the Golgi complex. In HCMV infected cells **(B)**, the nuclear egress complex is in the dotted line box. In contrast, VAC is found in the infected cells next to the nuclear and indicated by the dotted line box **(C)**, at a magnification of 20,000, 40,000, and 80,000, respectively.

Under TEM, the cell boundary and the nucleus with bilayer membrane structure were distinguished. After step-by-step amplification, the organelles of HFF can be identified in the cytoplasm, which is involved in metabolism and biosynthesis, such as the ribosome, mitochondria, endoplasmic reticulum, and Golgi body. The nuclear bilayer membrane structures, autosomes, and chromosomes can be observed, too (Figure 3A). Substantial amounts of lysosomes accumulated in the cytoplasm of the HCMV infected HFF cells (Figure 3B). Nucleocapsids are formed by DNA and capsid proteins within the host cell nucleus. Egression of nucleocapsids through nuclear egress complex (NEC) and the VAC was observed in the infected HFF cells, too (Figures 3B,C).

**FIGURE 4:**
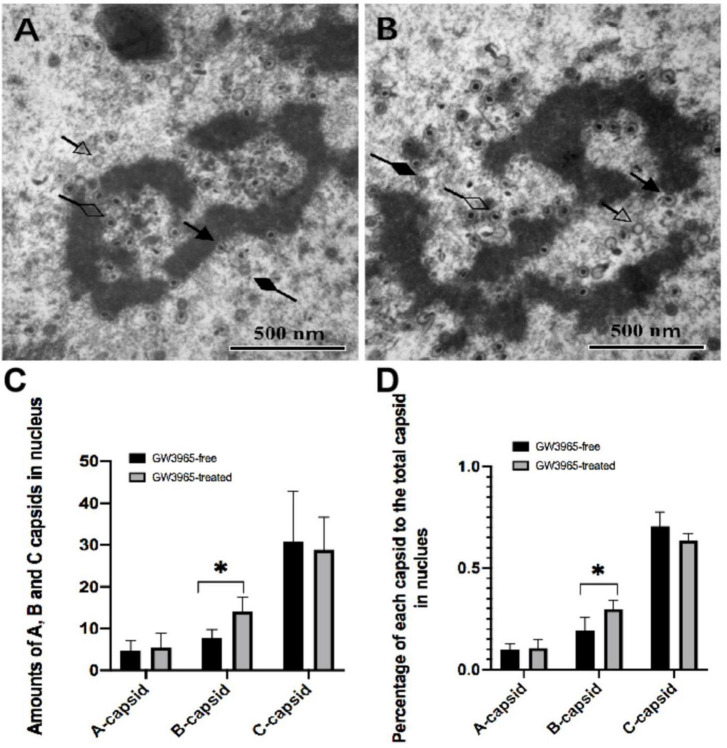
Numbers of the three kinds of capsids in the nucleus of cells treated with and without GW3965. **(A,B)** The solid arrow points to the capsid undergoing DNA encapsidation. The capsids pointed by a hollow arrow, solid diamond-head arrow, and hollow diamond-head arrow are A-capsids, B-capsids, and C-capsids, respectively, at a magnification of 40,000. The numbers and the percentages of each kind of capsid in the nucleus of cells treated with and without GW3965 are presented in panels **(C,D)**, respectively *P < 0.05.

DNA encapsidation is a step in which viral DNA is packed into the capsid, resulting in three types of virus nucleocapsids in the nucleus. Among them, A-capsids that appear empty, lacking either scaffold of immature capsids or packaged viral DNA of mature nucleocapsids, is believed to result from abortive attempts to package virus DNA. B-capsids that contain scaffold but no viral DNA may include precursors to nucleocapsids because they accumulate when viral DNA packaging is blocked in HCMV. C-capsids contain viral DNA without any scaffold and thus probably represent nucleocapsids in the process of maturation. To evaluate the effects of activated LXR on viral DNA encapsidation, numbers of the three kinds of capsids in the nucleus of the infected cells treated without (Figure 4A) and with (Figure 4B). GW3965 were counted in seven randomly selected nucleus regions of the TEM pictures with the same magnification, respectively.

No significant differences of quantities as well as percentage of A and C capsids were found between infected HFF treated with and without GW3965 (Figures 4C,D). The result indicates that activated LXR has no effects on viral DNA encapsidation of A-capsids and C-capsids. However, activated LXR increases the number of B-capsids. The percentage of B-capsid to total capsids was 19.4% in the GW3965-free group and was 29.8% in the GW3965-treated group (*P* < 0.05).

### Effects of Activated Liver X Receptor on Virion Maturation

**FIGURE 5:**
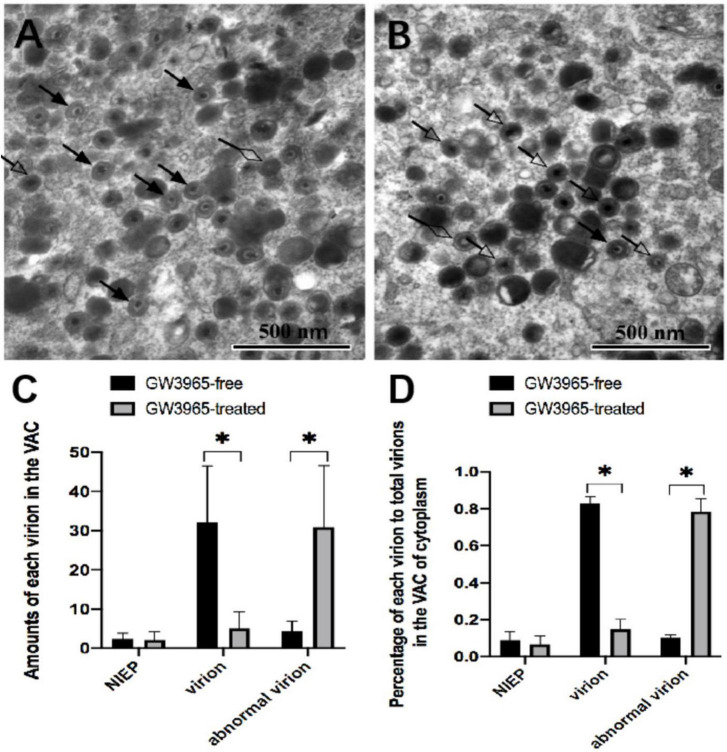
Effects of activated LXR on virion maturation in the cytoplasm of infected cells. NIEP, virions, and abnormal virions can be found in panels **(A,B)**. The hollow diamond-head arrow points to the non-infectious enveloped particle. The solid arrow indicates the infectious virion. The hollow arrow shows the abnormal virion containing the viral genome but no intact bilayer membrane structure at a magnification of 40,000. The numbers and the percentages of each type of virions in VAC of infected cells treated with or without GW3965 are presented in panels **(C,D)**, respectively. Data were analyzed by the *t*-test *P < 0.05.

In HCMV infected HFF cells, dense body, non-infectious enveloped particle (NIEP) and virion were seen in the VAC region of infected cells. To evaluate the effects of activated LXR on virion maturation, VAC regions were randomly selected, and the numbers of each kind of virion in VAC were counted. The defective form of progeny virus particles was described as abnormal virion in this study. Those abnormal virions can be clearly seen (Figure 5B). Although they contain DNA, the envelopes are blurred, making it difficult to distinguish the complete and clear membrane structure.

Compared to those in infected HFF treated without GW3965 (Figure 5A), significantly higher amounts (Figure 5C) and percentage (Figure 5D) of abnormal virions and lower amounts and percentage of normal virions to total virions were found in the VAC of infected HFF treated with GW3965 (Figure 5B). Activated LXR decreased the percentage of normal virus particles in VAC from 83 to 15% (*P* < 0.05), while abnormal virus particles increased from 10 to 78% (*P* < 0.05). The result demonstrated that activated LXR significantly increased the abnormal virions and reduced the normal virions in HCMV infected HFF cells. Activated LXR causes abnormalities in the cytoplasmic membrane, which also causes abnormalities in the assembly process of progeny viruses.

## Discussion

During HCMV maturation, viral capsid assembly is assisted by a pUL80-based scaffold and the viral maturational protease pUL80a (Cardone et al., 2012). The nucleocapsids are translocated from the nucleus to the VAC in the cytoplasm via an NEC (Gibson, 2008; Liu et al., 2011). In VAC, the virus acquires its remaining tegument and the viral envelope through secondary envelopment before egressing from the infected cells. The success or failure of DNA packaging determines the fate of the capsids and results in three types of virus nucleocapsids in the nucleus. The processes of HCMV maturation also include envelopment and de-envelopment (Das and Pellett, 2011). Intranuclear capsids bud through the inner nuclear membrane via a primary envelopment process (Indran and Britt, 2011). De-envelopment occurs at the outer nuclear membrane to release the nucleocapsid into the cytoplasm, where it acquires the final complement of tegument protein (Moorman et al., 2010). Fully tegument capsids bud into cytoplasmic vesicles or tubules, the capsids undergoing secondary envelopment in the cytoplasm (Meissner et al., 2012). Activated LXR seems to affect various stages of progenitor virus assembly and maturation, but which part plays a key role remains to be studied further.

Initiation of HCMV replication depends on transcription from the viral major immediate-early (IE) gene. The IE proteins are believed to control all subsequent early and late events in HCMV replication, including reactivation from latency, in part by antagonizing intrinsic and innate immune responses. The activated LXR significantly reduced IE86 in our study. Moreover, activated LXR decreased the expression of the pp28 and pp65 which are both the main tegument protein of the virus, and envelope glycoprotein gB. Tegument proteins are important for many processes throughout infection, including disassembly of virions, modulation of cellular responses, and virion maturation. Activated LXR significantly reduced the expression of several important tegument proteins, which may be an essential part for inhibiting the maturation of virus particles. However, the activated LXR did not almost inhibit the pp52 protein. HCMV pp52 acts as the accessory subunit of DNA polymerase, whose role is to increase the process of polymerization, plays an essential role in viral DNA replication. We found that DNA copies in virions with the treatment of GW3965 were only decreased approximately 2.5-fold, similar to those of the infected HFFs activated by LXR (our unpublished data). The minimal effect on the expression of pp52 activated by LXR suggested that it might be one reason explaining that viral DNA synthesis had no obvious decline by the treatment with GW3965.

Cholesterol is the main component of the viral envelope structure (Wofford et al., 2020). The envelope mediates the fusion of the virus to host cells after absorption by a viral receptor (Muranyi et al., 2002; Hasan et al., 2018). Activated LXR can reduce quantities of cholesterol in HCMV infected fibroblasts and progeny virions. Lacking cholesterol during virus assembly and maturation may be an important reason for defective virus particles.

Based on TEM technology, this study can intuitively understand the morphological changes of progeny virus affected by LXR activation and preliminarily explore the key positions and stages of progeny virus defects (Krzyzaniak et al., 2009; Klupp et al., 2017). The nucleus was observed under TEM and three viral capsids were counted. A- and B-capsids are thought to be either intermediates formed during nucleocapsid assembly or abortive forms that fail to undergo DNA encapsidation. Activated LXR had no effect on A-capsids but increased the number of B-capsids. B-capsids that are successfully filled with viral DNA transition into C-capsids and may further mature into virions. We found that the synthesis process of C-capsids, which eventually form normal virions in the nucleus, was not affected by the activation of LXR.

The endosomal membranes are responsible for envelopment with the exchange of cellular organelle membranes in character during HCMV infection (Mach et al., 2005; Tandon et al., 2009). Trans-Golgi network (TGN) and endoplasmic reticulum (ER)-Golgi intermediate compartments (ERGIC) also contribute to HCMV envelopment (Calistri et al., 2007; Wallis et al., 2021). Similarly, viral proteins are predicted to employ several independent cellular pathways to accumulate into VAC during later stages of viral morphogenesis (Crump et al., 2007; Jean Beltran et al., 2016). Corresponding to various nucleocapsids, three different viral particles are formed in the cytoplasm during HCMV infection, including dense bodies which are non-infectious particle carrying pp65 tegument protein, NIEP, and virion that are produced from B-capsids and C-capsids, respectively. When the viral nucleocapsid passes through the nuclear membrane and reaches the VAC, the virus particles have significant changes in morphology. There were many defective virus particles in VAC activated by LXR. Compared with normal virus particles, the nucleocapsid structure of the abnormal virions is relatively regular, but the envelope structure is fuzzy, and the boundary between the layers of the envelope is not clear. The complex envelope structure outside the nucleocapsid of the virus is mainly composed of tegument protein and organelle membrane. Activation of LXR might affect the organelle membrane structure by inhibiting cholesterol synthesis in cells, resulting in defects in virus particles. Therefore, we hypothesized that activation of LXR mainly inhibited the maturation of viral assembly in the cytoplasm of infected cells.

Our study found that activated LXR might affect organelle membrane structure by inhibiting cholesterol synthesis in cells, reducing the envelope integrity of maturating virions. Thus, there were much more defective virus particles that were produced at the VAC mediated envelopment during the assembly and maturation, resulting in the decrease of progeny virulence. However, the mechanism by which activated LXR inhibits the maturation of HCMV virion particles remains to be explored.

## Data Availability Statement

The raw data supporting the conclusions of this article will be made available by the authors, without undue reservation.

## Author Contributions

YQ and QR conceived and designed the study. BL performed the experiments and wrote the manuscript. BL, YM, YH, and ZL analyzed the data, contributed reagents, materials, and analysis tools. All authors read and approved the final manuscript.

## Conflict of Interest

The authors declare that the research was conducted in the absence of any commercial or financial relationships that could be construed as a potential conflict of interest.

## Publisher’s Note

All claims expressed in this article are solely those of the authors and do not necessarily represent those of their affiliated organizations, or those of the publisher, the editors and the reviewers. Any product that may be evaluated in this article, or claim that may be made by its manufacturer, is not guaranteed or endorsed by the publisher.
